# Sleep deprivation-induced multi-organ injury: role of oxidative stress and inflammation

**DOI:** 10.17179/excli2015-245

**Published:** 2015-05-18

**Authors:** Srinivasan Periasamy, Dur-Zong Hsu, Yu-Hsuan Fu, Ming-Yie Liu

**Affiliations:** 1 Department of Environmental and Occupational Health, College of Medicine, National Cheng Kung University, Tainan 70428, Taiwan

**Keywords:** sleep deprivation, multi-organ injury, inflammation, oxidative stress

## Abstract

Sleep deprivation affects all aspects of health. Adverse health effects by sleep deviation are still underestimated and undervalued in clinical practice and, to a much greater extent in monitoring human health. We hypothesized that sleep deprivation-induced mild organ injuries; oxidative stress and inflammation might play a crucial role in inducing multi-organ injury. Male C57BL/6J mice (n = 6-7) were sleep-deprived for 0-72 h using a modified multiple platform boxes method. Blood and tissue were collected. Liver, heart, kidney, lung, and pancreatic injuries were evaluated using biochemical and histological analyses. Glutamic oxaloacetic transaminase (GOT), glutamic pyruvic transaminase (GPT), total billirubin (TBIL), creatine phosphokinase (CPK), creatine phosphokinase-myocardial band (CKMB), lactic dehydrogenase (LDH), creatinine (CRE), and blood urea nitrogen (BUN) were assayed in blood. Malondialdehyde (MDA), nitric oxide (NO), tumor necrosis factor (TNF)-α, interleukin (IL)-1β, and IL-6 levels were measured. Histology revealed mild-to-moderate liver and lung injury in sleep-deprived mice. Sleep-deprived mice had significantly higher GOT, GPT, TBIL, CPK, CKMB, LDH, BUN, and α-amylase (AMYL) levels, which indicated liver, heart, kidney, and pancreatic injuries. Serum IL-1β at 24 h and IL-6 at 72 h were significantly higher in sleep-deprived than in control mice. Hepatic TNF-α and IL-1β were significantly higher, but IL-6 significantly lower in mice that had been sleep-deprived for 72 h. Sleep deprivation-mediated inflammation may be associated with mild to moderate multi-organ damage in mice. The implication of this study indicates sleep deprivation in humans may induce multi-organ injury that negatively affects cardiovascular and gastrointestinal health.

## Introduction

Sleep has important functions for every organ in the body, and sleep deprivation leads to disorders that cause irreparable damage (Lima et al., 2014[26]). Sleep is a restorative process that plays an important role in the balance of psychological and physical health. Sleep loss may be associated with adverse health effects such as obesity, type 2 diabetes, hypertension, and cardiovascular disease (Grandner et al., 2014[20]; Guo et al., 2013[21]). Sleep duration among American adults has decreased significantly over the past 25 years. A rapid increase in the percentage of adults report an average sleep duration of ≤ 6 h/day (Luckhaupt et al., 2010[27]). Reduction in sleep duration and sleep quality is progressively common in modern society and is likely linked to changes in the socio-economic environment and lifestyle (Bixler, 2009[4]). The percentage of adults reported sleeping 6 h or less increased by 5 % - 6 % between 1985 and 2004 (NSF, 2005[33]). However, both short and long habitual sleep loss are associated with an increased risk of mortality (Gangwisch et al., 2008[16]), hypertension (Gangwisch et al., 2006[17]), coronary heart disease (Ayas et al., 2003[2]), and diabetes (Gangwisch et al., 2007[18]). Sleep deprivation in humans and rats show increased food intake (Martins et al., 2010[29]; Galvão et al., 2009[14]). However, sleep deprived animals show intense catabolism (Hipolide et al., 2006[22]) and energy expenditure, resulting in weight loss during the sleep deprivation period (Koban and Stewart, 2006[25]). In addition, short sleep duration is associated with self-rated poor health (Steptoe et al., 2006[47]) and elevated body mass index (BMI) (Taheri et al., 2004[48]).

Oxidative stress is as an imbalance between the formation and elimination of reactive oxygen/nitrogen species and is associated with several adverse outcomes such as cancers, immunodeficiency diseases, neurological diseases, and cardiovascular diseases (Turrens, 2003[49]). Furthermore, it is involved in the mechanisms of aging, pathogenesis of cancer, atherosclerosis, diabetes, and neurodegenerative disorders (Droge, 2002[9]). Free radicals accumulate during waking as a result of enhanced metabolic activity and are responsible for the effects of sleep deprivation (Reimund, 1994[43]). Sleep deprivation in animals and obstructive sleep apnea syndrome in human are also associated with increased oxidative stress (McEwen, 2006[30]; Barcelo et al., 2006[3]).

Inflammation is a type of non-specific immune response that functions by directing components of the immune system to the site of injury. Inflammation can be persistently activated in response to disease and genetic predisposition, etc. Insufficient sleep can provoke inflammation response via increased cytokine secretion (Vgontzas et al., 1999[50]). Cytokines are associated with sleep, including IL-1β, TNF-α, and IL-6 (Opp, 2005[34]). The immune system alters during the day along with the sleep-wake cycle. Immune cells in the blood are increased in the early evening and decreased in the morning (Redwine et al., 2004[41]). Cytokines serve as chemical messengers to attract and direct other components of the immune system are also at their highest levels at night (Redwine et al., 2000[42]; Born et al., 1997[5]). Disruption of the normal sleep wake cycle via sleep deprivation can affect immune function in humans (Simpson and Dinges, 2007[45]).

The focus of the present study was to evaluate sleep deprivation-induced multi-organ injury and the role of oxidative stress and inflammation in mice by the modified multiple platform method. Our current animal model was not considered a replication of typical real-life human sleep deprivation. However, transmeridian flight crews (Bradley and Floras, 2003[6]) and deep-sea fishing industry workers (Gander et al., 2008[20]) tend to work for more than 3 days with minimal sleep. Nonetheless, it is worth pointing out that 3 days of complete sleep deprivation is unheard of in patients in less than critical condition. However, it is common that the critically ill can have a near total loss of slow-wave sleep, rapid eye movement (REM) sleep, or both, for as long as 5-14 days (Orr and Stahl, 1977[35]). Therefore, we studied the effect of sleep deprivation on multi-organ injury associated oxidative stress and inflammatory indicators in mice. 

## Materials and Methods

### Reagents 

All the chemicals used in this study were purchased from Sigma-Aldrich (St. Louis, MO).

### Animals 

Male C57BL/6J mice 7-8 weeks old and weighing 25-30 g were purchased from our institution's Laboratory Animal Center. They were given a pellet feed diet and water *ad libitum*. They had a 12-h light/dark cycle and central air conditioning (25 °C, 70 % humidity) throughout the experiment. The animal care and experimental protocols were in accordance with nationally approved guidelines (No. 102122).

### Experimental protocols

A modified multiple platform method was used, which uses a REM technique to manipulate sleep deprivation, to actuate sleep deprivation in mice. An acrylic tank (40 x 30 cm) with 12 columns (platforms, 5 x 3 cm) was filled with 1 cm water. Five mice, all from the same cage, were placed in each tank for 24, 48, and 72 h, with water and food *ad libitum*. The loss of muscle tone associated with sleep deprivation caused them to touch the water and wake up. This model does not impose restriction of movement or social isolation (Patti et al., 2010[37]). Sleep deprivation for 24 h is designated as SD1, for 48 h as SD2, and for 72 h as SD3.

### Blood collection

The mice were given a light ethyl ether anesthesia, after which blood samples were collected. Blood was drawn via venipuncture into a serum separation tube, allowed to clot for 20-30 min at room temperature, and then centrifuged at 15000 rpm at 4 °C for 15 min.

### Assessing organ dysfunction and injury

Organ dysfunction and injury were assessed using a blood biochemical analyzer (DRI-CHEM 3500s; Fujifilm, Kanagawa, Japan) that measured serum levels of glutamic oxaloacetic transaminase (GOT), glutamic pyruvic transaminase (GPT), total billirubin (TBIL), creatine-phospho-kinase (CPK), creatine phosphokinase-MB (CKMB), lactic dehydrogenase (LDH), creatinine (CRE), and blood urea nitrogen (BUN).

### Histology and scoring system

Samples of liver, lung, heart, kidney, and pancreatic tissue from the mice were cut and placed in 10 % formalin. The samples were dehydrated using a graded percentage of ethanol and then fixed in paraffin wax for 1 h to form blocks. The blocks were trimmed and cut into 4-µm thick sections, stained with hematoxylin and eosin (H&E), and then mounted using Depex-Polystyrene dissolved in xylene mountant. The tissue sections were examined under a microscope (magnification: 100x) to assess pathology.

Four-to-six tissue sections per mouse were evaluated at both high and low power. Pathology scores of 1-5 were based on the percentage of tissue affected: 1 = 0 %, 2 = 1-25 %, 3 = 26-50 %, 4 = 51-75 %, and 5 = 76-100 %. Categories included interstitial changes (interstitial or interalveolar septal thickening), inflammation (intra-alveolar neutrophilic infiltrate), and consolidation (a combination of both cellular debris and fibrin-filled alveolar space) (Srinivasan and Liu, 2012[46]). Liver injury was scored using a slightly modified protocol: 1 = 0 %, 2 = 1-10 %, 3 = 11-20 %, 4 = 21-30 %, and 5 = 31-40 % (Srinivasan and Liu, 2012[46]; Periasamy et al., 2011[39]).

### Measuring nitric oxide content 

Briefly, the amount of nitric oxide (NO) in liver tissue was measured after the Griess reaction. Liver tissue was homogenized in deionized water (1:10, wt/vol). Tissue homogenate (500 L) was centrifuged at 2500 *g *for 10 min at 4 °C. Supernatant (100 L) was incubated with 100 L of Griess reagent at room temperature for 20 min. The absorbance was measured at 550 nm using the spectrophotometer. NO concentration was calculated by comparing it with a standard solution of known sodium NO concentration.

### Measuring lipid peroxidation levels

Liver tissue was homogenized in Tris HCl (20 mmol/L; pH 7.4). Tissue homogenate (500 L) was centrifuged at 2500 *g *for 10 min at 4 °C, and the supernatant (200 L) was measured at 586 nm for lipid peroxidation (Lipid Peroxidase Assay Kit; Calbiochem-Novabiochem, Darmstadt, Germany) using the spectrophotometer.

### Measuring TNF-a, IL-1β, and IL-6 levels

Tissue was homogenized in deionized water (1:10; wt/vol) and centrifuged at 1250 *g *for 10 min at 4 °C. The TNF-α, IL-1β, and IL-6 levels in the tissue supernatant were determined using an enzyme-linked immunosorbent assay (ELISA) (R&D Systems, Minneapolis, MN). TNF-α, IL-1β, and IL-6 were assessed by measuring absorbance at 450 nm and extrapolating from a standard curve with a sensitivity limit of 32.5 pg/mL. Protein concentration (pg/mg) in liver tissue was determined using protein assay dye (Bio-Rad Laboratories, Hercules, CA).

### Statistical analysis 

All statistical analyses were done using SPSS 11.0.1 (SPSS Inc., Chicago, IL). Data are means ± standard deviation (SD). Differences in the measured variables between each group were assessed using Fisher's Least Significant Difference (LSD) test. Significance was set at *P* < 0.05.

## Results

### Serum IL-1β, IL-6, and NO levels in sleep-deprived mice

**Figure 1:**
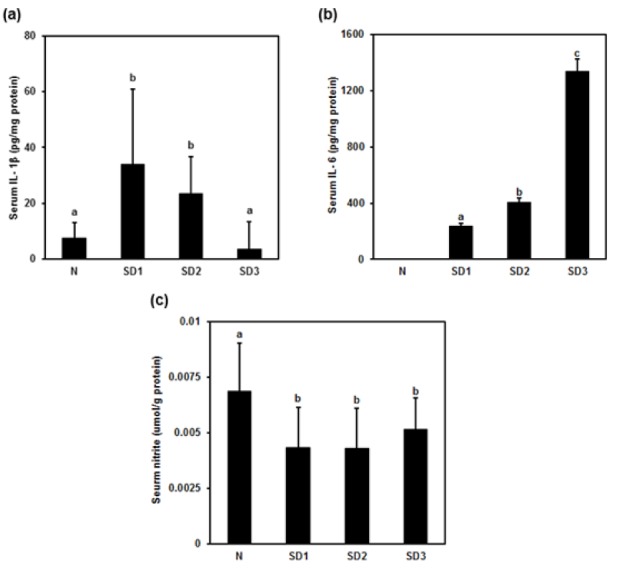
Effect of sleep deprivation on serum cytokines and nitric oxide (NO). N: control mice (not sleep-deprived); SD1: mice sleep-deprived for 24 h; SD2: mice sleep-deprived for 48 h; SD3: mice sleep-deprived for 72 h. (a) serum interleukin (IL)-1β; (b) serum IL-6; (c) serum NO. Data are means ± SD. ^a,b,c ^The differences between treatments with different letters are significant (*P* < 0.05).

Serum IL-1β was significantly higher in mice deprived of 24 h of sleep (SD1) than in controls (N). The changes in IL-1β levels were time dependent: IL-1β levels in mice deprived of 72 h of sleep (SD3) were nonsignificantly lower than in controls (Figure 1a(Fig. 1)). The changes in serum IL-6 levels were also time dependent: IL-6 was nondetectable in controls and highest in SD3 group mice (Figure 1b(Fig. 1)). Serum NO was significantly lower in all three SD groups than in controls (Figure 1c(Fig. 1)).

### Liver injury, cytokines, LPO, and NO levels

**Figure 2:**
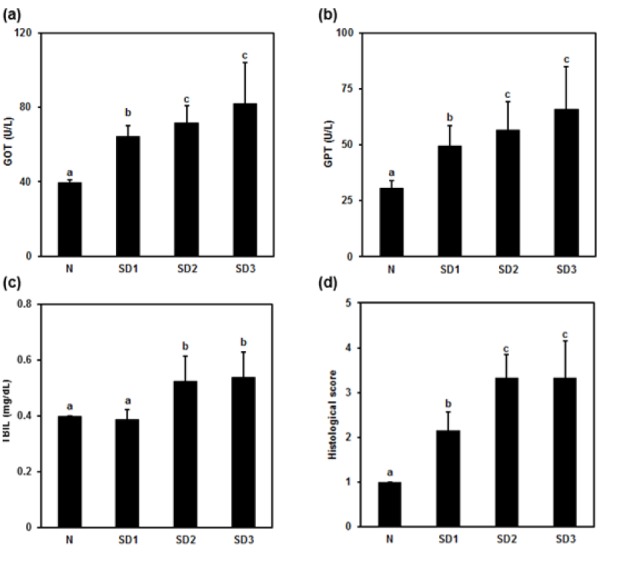
Effect of sleep deprivation on liver markers. (See groups and treatment details in legend for Figure 1). (a) glutamic oxaloacetic transaminase (GOT); (b) glutamic pyruvic transaminase (GPT); (c) total billirubin (TBIL); (d) histological score. Data are means ± SD. ^a,b,c ^The differences between treatments with different letters are significant (*P* < 0.05).

Serum GOT, GPT, and TBIL were, except for the TBIL level in the SD1 group, significantly and time-dependently higher in SD group mice than in controls (Figure 2a-c(Fig. 2)). There was no significant difference between the SD2 and SD3 groups in GOT, GPT, or TBIL levels.

**Figure 3:**
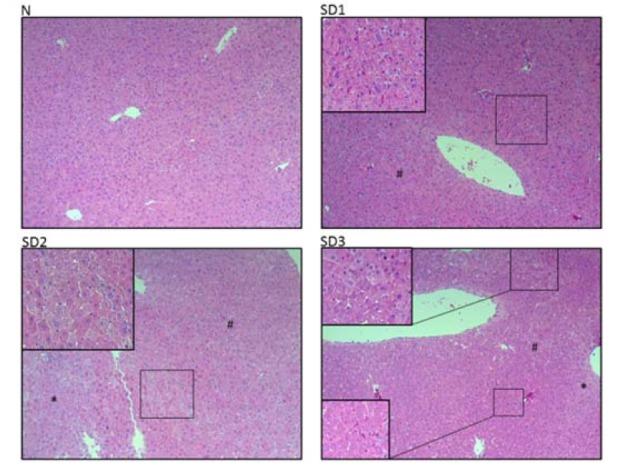
Effect of sleep deprivation on liver histology. (See groups and treatment details in legend for Figure 1). Photomicrographs of liver histology at [10x] x [10x]

SD1 group mice showed a mild morphological change in hepatocytes. SD2 and SD3 group mice showed mild necrotic hepatocytes around the central and portal vein. Few atypical hepatocytes with cytoplasmic enlargement and increased nuclear density were observed. Hepatocytes exhibited mild-to-moderate swelling or ballooning, pale cytoplasm, and few lytic necrosis (Figures 2d(Fig. 2) and 3(Fig. 3)).

**Figure 4:**
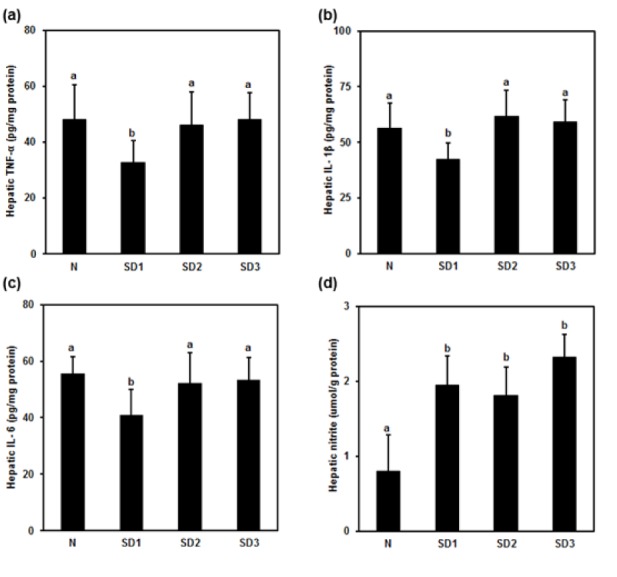
Effect of sleep deprivation on liver cytokines and NO. (See groups and treatment details in legend for Figure 1). (a) Tumor necrosis factor (TNF)-α; (b) IL-1ß; (c) IL-6; (d) NO. Data are means ± SD. ^a,b ^The differences between treatments with different letters are significant (*P* < 0.05).

TNF-α (Figure 4a(Fig. 4)), IL-1β (Figure 4b(Fig. 4)), and IL-6 (Figure 4c(Fig. 4)) were significantly lower in SD1 group mice than in controls, but the differences between SD2, SD3, and control group mice were non-significant.

Hepatic LPO levels in SD group mice were not significantly different from those in controls (Table 1(Tab. 1)). NO levels were significantly higher in the SD groups than in the control group. The differences between the SD groups were not significant (Figure 4d(Fig. 4)).

### Lung injury, cytokines, LPO, and NO levels

**Figure 5:**
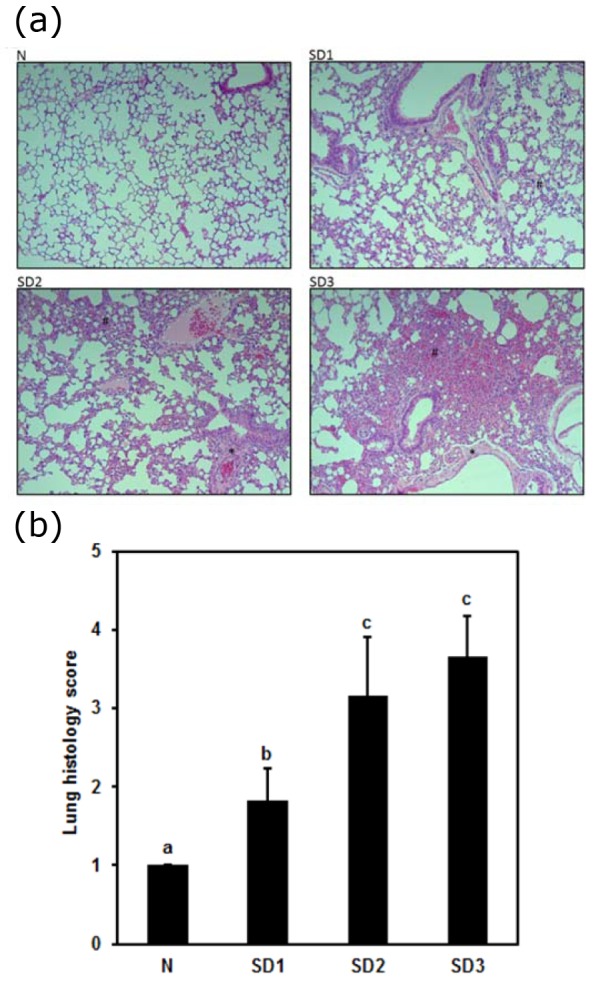
(a) Effect of sleep deprivation on lung histology. (See groups and treatment details in legend for Figure 1). Photomicrographs of liver histology at [10x] x [10x] (b) Effect of sleep deprivation on lung histology score. (See groups and treatment details in legend for Figure 1). Data are means ± SD. ^a,b,c^The differences between treatments with different letters are significant (*P* < 0.05).

Lung pathology showed more histological evidence of lung injury mild-to-moderate interstitial thickening, and cellular infiltration in the interstitium and alveolar compartments in SD group mice than in control group mice. In addition, SD3 group mice showed greater interstitial thickening and thickening of the bronchial cartilage (Figure 5a(Fig. 5)). Lung histological scores were significantly higher in the SD groups than in the control group, SD2 and SD3 group scores were significantly higher than were SD1 group scores (Figure 5b(Fig. 5)).

**Figure 6:**
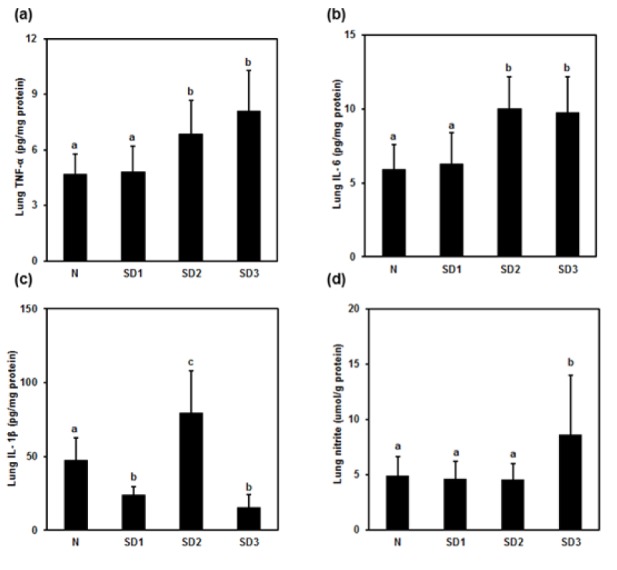
Effect of sleep deprivation on lung cytokines and NO. (See groups and treatment details in legend for Figure 1). (a) TNF-α; (b) IL-1ß; (c) IL-6; (d) NO. Data are means ± SD. ^a,b,c^The differences between treatments with different letters are significant (*P* < 0.05).

TNF-α (Figure 6a(Fig. 6)) and IL-6 (Figure 6b(Fig. 6)) levels were significantly higher in SD2 and SD3 group mice than in control group mice. IL-1β (Figure 6c(Fig. 6)) was significantly higher in SD2 group mice than in control group mice. 

In SD1 and SD3 group mice, however, IL-1β levels were significantly lower than in control group mice. NO levels were significantly different (higher) only in the SD3 group (Figure 6d(Fig. 6)). The differences in LPO levels between all four groups were nonsignificant (Table 1(Tab. 1)).

### Myocardial injury, cytokines, malondialdehyde (MDA), and NO levels

**Figure 7:**
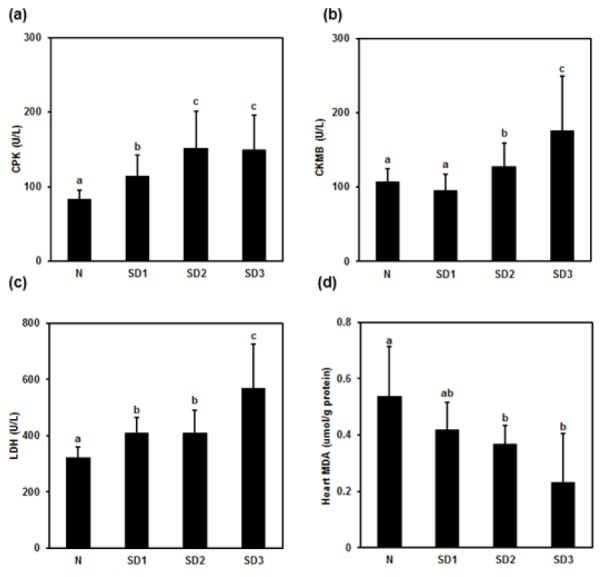
Effect of sleep deprivation on heart markers and MDA. (See groups and treatment details in legend for Figure 1). (a) Creatine phosphokinase (CPK); (b) creatine kinase myocardial band (CKMB); (c) lactic dehydrogenase (LDH); (d) malondialdehyde (MDA) (^“ab”^ above SD1 bar means “no difference when compared with ^'a'^ or ^'b'^”). Data are means ± SD. ^a,b,c^The differences between treatments with different letters are significant (*P* < 0.05).

Serum creatine phosphokinase (CPK) was significantly and time-dependently higher in the SD group mice than in the controls. The difference between the SD2 and SD3 groups was nonsignificant (Figure 7a(Fig. 7)). Serum creatine kinase myocardial band (CKMB) levels were significantly higher in the SD2 and SD3 group mice than in the controls; however, they were nonsignificantly lower in SD1 group mice than in control mice (Figure 7b(Fig. 7)). Serum LDH was significantly and time-dependently higher in SD group mice than in controls, and significantly higher in SD3 group mice than in SD1 and SD2 group mice (Figure 7c(Fig. 7)). Heart MDA levels were significantly lower in SD2 and SD3 mice than in SD1 and control mice (Figure 7d(Fig. 7): ^“ab”^ above SD1 bar means “no difference when compared with ^'a'^ or ^'b'^”).

**Figure 8:**
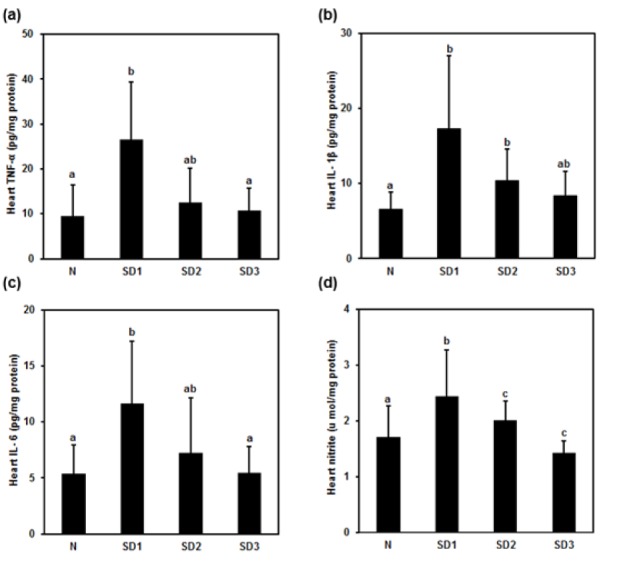
Effect of sleep deprivation on heart cytokines and NO. (See groups and treatment details in legend for Figure 1). (a) TNF-α; (b) IL-1ß; (c) IL-6; (d) NO. Data are means ± SD. ^a,b,c^The differences between treatments with different letters are significant (*P* < 0.05). (^“ab”^ above SD2 bar in (a) and (c) and above SD3 bar in (b) means “no difference when compared with ^'a'^ or ^'b'^”).

Heart TNF-α and IL-1β levels were significantly higher only in SD1 and SD2 group mice than in control mice (Figure 8a, b(Fig. 8): ^“ab”^ above SD2 bar in (a) and above SD3 bar in (b) means “no difference when compared with ^'a'^ or ^'b'^”). IL-6 was significantly higher in SD1 group mice (Figure 8c(Fig. 8)). Heart NO levels were significantly higher only in SD1 group mice and significantly lower in SD3 mice (Figure 8d(Fig. 8)).

### Serum BUN, LPO, NO, TNF-α, and IL-6 levels 

**Figure 9:**
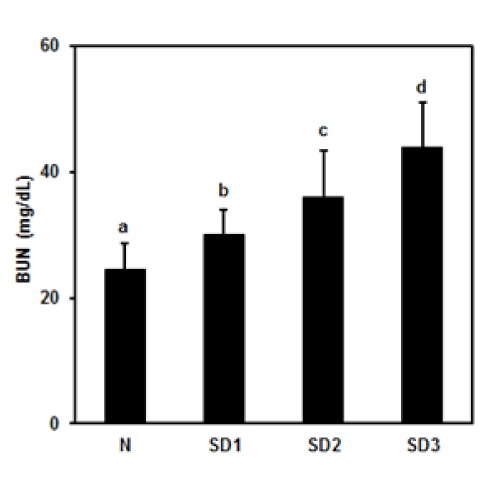
Effect of sleep deprivation on kidney marker. (See groups and treatment details in legend for Figure 1). Blood urea nitrogen (BUN). Data are means ± SD. ^a,b,c^The differences between treatments with different letters are significant (*P* < 0.05).

Serum BUN levels were time-dependently and significantly higher in the SD group mice (Figure 9(Fig. 9)) than in the controls, but there were no significant differences in serum LPO, NO, TNF-α, or IL-6 levels between the four experimental groups (Table 1(Tab. 1)).

### Spleen TNF-α, IL-1β, and NO levels

**Figure 10:**
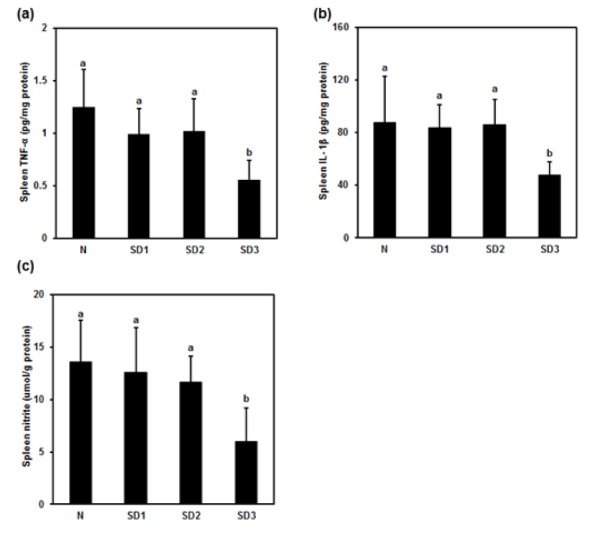
Effect of sleep deprivation on spleen cytokines and NO. (See groups and treatment details in legend for Figure 1). (a) TNF-α; (b) IL-1ß; (c) NO. Data are means ± SD. ^a,b^The differences between treatments with different letters are significant (*P* < 0.05).

Spleen TNF-α (Figure 10a(Fig. 10)) and IL-1β (Figure 10b(Fig. 10)) were significantly lower in SD3 group mice than in control mice. However, TNF-α and IL-1β levels in the SD1 and SD2 groups were not significantly different from those in the control group, nor were IL-6 levels between the four experimental groups significantly different (Table 1(Tab. 1)).

NO levels were significantly lower in SD3 mice, but not in SD1 or SD2 mice, than in control mice (Figure 10c(Fig. 10)). There were no significant differences in spleen LPO levels between the experimental groups (Table 1(Tab. 1)).

## Discussion

Sleep deprivation-induced moderate multi-organ injury through oxidative stress and inflammation in mice. Sleep deprivation increased serum GOT, GPT, and TBIL indicating liver injury. Liver cytokines were altered in sleep deprived mice. In addition, sleep deprivation increased nitrite level. Serum CPK, CKMB, and LDH were increased demonstrating myocardial injury. Myocardial TNF-α, IL-1β, and IL-6 were increased in initial 24 h and subsequently decreased in 72 h of sleep deprivation. Myocardial oxidative stress indicated by MDA and NO were decreased on sleep deprivation. BUN increased signifying kidney dysfunction. Sleep deprivation increased lung edema, nitrite, TNF-α, IL-1β, and IL-6 level. In addition, it decreased spleen nitrite, TNF-α, and IL-1β level indicating immune compromise.

Sleep deprivation-induced liver injury. Sleep deprivation increased GOT, GPT, and TBIL. In addition, it increased hepatic nitrite; however, no alterations in the level of lipid peroxidation. Sleep deprivation induced mild morphological change in the liver. Mild necrotic hepatocytes were observed around central and portal vein. In addition, cytoplasmic enlargement with increased nuclear density and mild swelling or ballooning of hepatocytes was found in sleep deprived animals. Time (24-72 h) dependent increase in GOT, GPT, and TBIL in sleep deprived animals indicated mild liver injury. Inflammatory cytokines decreased at 24 h and back to normal in 48 and 72 h sleep deprivation. However, nitric oxide increased in all sleep deprived animals. 

Few previously published studies reported sleep deprivation may not cause oxidative damage, nor that can it represent an oxidative stress for the brain or for peripheral tissue such as liver and skeletal muscle (Gopalakrishnan et al., 2004[19]). In addition, sleep deprivation effects have not been localized to a specific tissue or system, and structural damage has not been observed in histopathology slides of major peripheral organs (Everson, 1993[11]). In contrary, it is also reported that peripheral cell membrane damage is an early consequence of sleep deprivation, relative to advanced morbidity and lethality (Everson et al., 2005[12]). Sleep deprived (72 h) male volunteers reported increased plasma AST and ALT level (Ilan et al., 1992[24]) indicating liver injury. Sleep deprivation at least partially mediated by reactive oxygen species (Lima et al., 2014[26]; Brown and Naidoo 2010[7]; Ramanathan et al., 2002[40]). In addition, it induces noxious metabolic and immunological alterations that eventually lead to lethal consequences; it is thought that anti-oxidant imbalance mediates these alterations. Elevated oxidative stress and insufficient antioxidant activities may result in liver cell injury (Lima et al., 2014[26]; Everson et al., 2005[12]).

Hepatic nitrite level increased, however, MDA level remain unaltered in sleep deprived mice. Therefore, no identified oxidative stress marker that directly linked oxidative stress and hepatic cell injury in cause-and-effect relationships (Everson et al., 2005[12]). In sleep deprived subjects, neutrophil migrates into interstitial spaces of organs signifies important biochemical alterations (Everson et al., 2008[13]). During tissue injury, mediators diffuse from the site of injury and activate the endothelium. Circulating phagocytes are activated, bind to endothelium, and pass out of the blood vessel dissolving the basement membrane. Neutrophils migrate into the tissues based on the strength of the chemotactic factors formed by alterations in the biochemistry of the tissues (Everson et al., 2008[13]). Oxidative stress may lead to cell death and also decreases non-enzymatic antioxidants in the cell, therefore the oxidative stress is not quenched, ultimately leads to oxidant damage (Everson et al., 2005[12]).

Sleep deprivation-induced lung and myocardial injury; altered inflammatory cytokines and oxidative stress parameters. Histology of lung revealed mild to moderate interstitial thickening, and cellular infiltration in the interstitium and alveolar compartments. Inflammatory processes are the etiological root of several medical evils. Therefore, inflammatory processes that may be induced by sleep deprivation are believed to have clinical and biological relevance, as well as potentially far-reaching implications (Everson et al., 2008[13]). Sleep deprivation has been demonstrated by increased pro-inflammatory cytokines, appetite, and blood pressure as well as cortisol levels (Copinschi, 2005[8]). It also leads to circadian rhythms disruption that has enormous implications in the pathogenesis of cardiac and renal disease (Martino et al., 2008[28]). Circadian rhythms play a pivotal role in the regulation of cardiovascular physiology. Disruption of diurnal rhythms increases mortality in cardiomyopathic hamsters (Penev et al., 1998[38]) and exacerbates pressure overload myocardial hypertrophy (Martino et al., 2008[28]). Diurnal cycling plays a key role in organ growth and renewal and disruption is a key contributor to disease (Martino et al., 2008[28]). In the present study, alterations in the pro-inflammatory cytokines and oxidative stress might play a role in the lung and myocardial injury in sleep deprived mice.

Sleep deprivation-induced renal dysfunction indicated by elevated BUN. Integrity of peripheral organs such as the heart and kidney depends on the circadian coordination. Long-term disruption of circadian rhythms, in shift workers, transoceanic flight attendants, or patients with sleep disturbances, can ultimately result in heart and kidney disease (Martino et al., 2008[28]). Circadian clocks provide temporal organization for the proliferation of renal tubular epithelial cells may give evidences about cortical cell apoptosis, and renal pathology (Martino et al., 2008[28]).

Sleep deprivation altered inflammatory cytokines and oxidative stress in spleen and serum. Sleep disruption have profound effects on the immune system. Alterations of the sleep wake cycle affect the number of circulating lymphocytes, natural killer cells, antibody titers, and levels of cytokines in humans (Mullington et al., 2009[32]; Hui et al., 2007[23]; Palma et al., 2006[36]; Everson, 2005[10]), and rodents (Palma et al., 2006[36]; Everson, 2005[10]; Renegar et al., 1998[44]), and increased inflammatory cytokines such as IL-6, C-reactive protein, and TNF-α (Mullington et al., 2009[32]; Vgontzas et al., 2004[51]; Meier-Ewert et al., 2004[31]) which translate into impaired immune function (Redwine et al., 2004[41]; Everson, 1993[11]). Sleep restriction in human was characterized by higher mitogen-stimulated levels of pro-inflammatory agents such as TNF-α and MCP-1, and a shift towards Th2 activity, as reflected by an altered Th1/Th2 cytokine balance (Axelsson et al., 2013[1]).

To conclude, sleep deprivation might induce multiple organ injury with altered cytokines and oxidative stress. Sleep deprivation in humans with static night shifts, flex shifts, extended shifts, rotating shifts, and frequent international travel by airline flight crews might undergo mild multiple organ injury which is undetected. Successive multi-organ injuries scar organs and induce fibrosis, which causes myocardial infarction, diabetes mellitus, and liver and kidney dysfunction. This might explain these chronic diseases in humans who undergo long-term successive sleep deprivation. 

## Notes

Srinivasan Periasamy and Dur-Zong Hsu contributed equally to this publication.

## Acknowledgements

This research was supported by grants NSC 99-2314-B-006-031-MY3 and NSC 102-2314-B-006-028-MY2 from the Taiwan Ministry of Science and Technology. The other authors have indicated no financial conflicts of interest.

## Figures and Tables

**Table 1:**
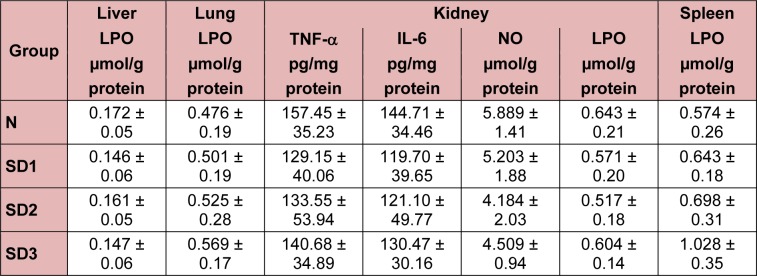
Parameters unaltered in sleep deprivation
